# Religion and mental health: an Eastern Mediterranean region perspective

**DOI:** 10.3389/fpsyg.2024.1441560

**Published:** 2024-08-07

**Authors:** Suhaila Ghuloum, Haya A. Q. F. Al-Thani, Hassen Al-Amin

**Affiliations:** ^1^Mental Health Services, Hamad Medical Corporation, Doha, Qatar; ^2^Weill Cornell Medicine, Doha, Qatar

**Keywords:** religion, mental health, Eastern-Mediterranean, attitude, possession, suicide, culture

## Abstract

In the last decade, spirituality gained increasing recognition, with an individualized approach to the significance of symptoms and the purpose of treatment. Many psychiatrists started adopting the term “biopsychosocial-spiritual” management. Modern psychological therapies have drawn from the principles of ancient Eastern traditions. However, the spiritual beliefs within Islam and their impact on mental well-being have yet to be studied sufficiently. The Eastern Mediterranean region is largely Muslim and shares similar cultural beliefs. This paper explores some of these beliefs and their impact on perceptions of mental health and help-seeking behavior. The paper will focus primarily on the religious ideologies, the concepts of punishment and reward from Allah (God) in the context of the high stigma associated with mental illness in the region, and the knowledge, attitudes, and perceptions toward people with mental illness. We will explore cultural beliefs in possession, evil eye, and black magic and how they influence treatment adherence. Traditional and faith healers remain very popular in this part of the world and are often the first line of treatment for people presenting with mental illness. Suicide and self-harming are particularly sensitive areas due to their religious implications in life and death, as well as the afterlife. Despite the global interest in the Eastern Mediterranean region, there still is a shortage of research on the intercultural aspects of this region, especially regarding mental health assessments. In this regard, we will explore some published literature from Qatar and draw comparisons with regional findings.

## Introduction/Background

The traditional biopsychosocial model of care in mental health held fort for many decades as the comprehensive approach to management, addressing biological, psychological, and social aspects of the patient’s conditions. However, in the last decade, the role of spirituality has gained momentum, with more research evidence supporting the positive impact of religiosity and spirituality on mental health outcomes (Labarta et al., 2024; Lynn and Schell, 2024). The terms (religiosity and spirituality) are often used interchangeably, with several definitions used to differentiate religion from spirituality (Arrey et al., 2016). This complicates the analysis of research evidence. There is a general agreement that Religion follows a set of recognized rules and systems shared by a group, while spirituality is an individualized experience aiming to answer questions about life and its meaning (Astrow et al., 2001; Lucchetti et al., 2021). However, from an Islamic perspective, religion and spirituality are the same and regulate all aspects of an individual’s life (Rassool, 2000; Heydari et al., 2016). With more literature evidence on the important role of spirituality in mental health outcomes, the model of care is now modified to “biopsychosocial-spiritual,” and spirituality is widely incorporated into undergraduate medical education curricula and psychiatry residency training programs (Hernandez et al., 2023).

In this paper, we will explore the interface between religion and mental health based on research findings from Qatar, compared with regional literature in the Eastern Mediterranean. The region is home to multiple faiths, the most prevalent of which is Islam, and is rich with cultural and historical beliefs often confused with religious ones. Yet, there are a few attempts to understand the topic better. In this regard, most of the relevant research from the Middle East focuses on cancer patients (Weathers, 2018), lacks interventional approaches, and utilizes tools validated on Western populations. In the following sections, we will address four common Islamic religious and mystical cultural aspects that interact with the assessment and treatment of patients with mental illness, namely: attribution of Islamic religion to knowledge and attitudes toward mental illness, the impact of religious convictions in magic and jinn causing abnormal behaviors, the role of faith healers and help-seeking behavior, and the relationship between suicidality and religious beliefs. The focus on these four aspects is based on their direct association with mental health care and their impact on patient outcomes, with more regional research addressing them (Al-Habeeb, 2003; El-Islam, 2009; Heydari et al., 2016; Amro et al., 2019, 2022; Arafat, 2022).

## Knowledge and attitudes

Islamic faith is viewed as a natural innate need to maintain well-being and to sustain the self against evil and harm. Illness, including the mental one, is seen as a test through which an individual must show the virtue of patience, relying on the strength of their faith. In this context, the worsening of illness is misinterpreted by those with mental illness (mainly their families) as a weakness in faith (Haque, 2004; Khullar, 2022), which delays seeking professional help. Precedents of refuge to faith and divinity are found in holy scriptures, where assessing faith and its strength becomes the initial step that sets the attitude toward help-seeking behaviors (Martinez et al., 2007; Bridi et al., 2023). Further, a paradoxical cultural conjecture exists in the Arab population, where illness is conceived as a form of punishment. In a survey of knowledge, attitudes, and beliefs about mental illness among the Arab population living in Qatar, almost half of the respondents (48.3%) believed mental illness could be a punishment from God; the vast majority of those were the younger generation aged 18–30 (Ghuloum et al., 2010). Exploring the biopsychosocial-spiritual model among patients treated for substance use in Jordan, addiction was believed to be associated with poor religious adherence to praying, fasting, and poor relationships with parents (Al-Ghaferi et al., 2017). Negative conceptualizations of mental illness as a punishment from God highlight the need for increasing mental health literacy and de-stigmatization.

Stigma toward mental illness is a global phenomenon. Still, it is also known to be more common in the Middle East and carries a complex social and religious component. Stigma toward mental health conditions is important to consider in the Arab region as it is a major predictor responsible for the underutilization of mental health services (Alluhaibi and Awadalla, 2022). Religious and cultural values are intertwined in this region, with many beliefs and practices wrongly attributed to religion. When considering stigmatization through Islamic constructs, patients’ religiosity influences their help-seeking behaviors. The religious stigma surrounding mental illness may undermine help-seeking and deter individuals from openly discussing their symptoms (Zolezzi et al., 2018). The World Health Organization reports a lower treatment rate for severe mental illness in the Middle East compared to the same conditions in other WHO-defined regions (World Health Organization, 2017). Stigma is not necessarily related to affluence or educational level; in the highly affluent Gulf Cooperation Council states, reluctance to seek mental health services and misperceptions about mental illness is very common, even among the highly educated (Al Saif et al., 2019). Patients’ religiosity may also influence preferences for gender-specific care, i.e., patients have to be assessed by staff of the same gender, particularly in conservative or traditional communities where interactions between unrelated men and women are restricted (Amro et al., 2019). The intersection between gender and religion also has important implications for help-seeking, whereby women may experience barriers to treatment to their status within society in addition to obstacles imposed by cultural traditions and religious misconceptions. Women with mental illness are less likely to be married, more likely to be divorced and abused, and therefore less likely to seek help or adhere to treatment. Furthermore, women are often the primary caregivers to other family members, including the elderly, and thus view their health needs as a lower priority (World Health Organization, 2000; Douki et al., 2007).

The interplay of societal beliefs and religiosity is intricate and not to be homogenized. Further understanding of this relationship is needed to aid in approaches and policies. A study among 16 Arab countries revealed that religiosity is associated with favorable attitudes toward help-seeking behaviors (Fekih-Romdhane et al., 2023a). This provides an opportunity for using appropriate religious constructs in demystifying and reforming socio-religious misconceptions of mental illness to promote health-seeking behaviors and empower against stigmatization. Muslims resort to religion when faced with difficult life situations, where they increase the frequency and depth of their religious activities like reading the Qur’an, praying, repenting, and submitting to God’s will. The Qur’an, the holy book of Islam, begins by introducing God as “The most compassionate, most merciful” (Al-Fatihah, 1:1) and proceeds to characterize God as a figure to seek help from and as the guide. This opening establishes the foundations of the Islamic faith by submitting to and relying on God. In line with the above, in a study among Iranian war veterans with post-traumatic stress disorder (PTSD), religious beliefs and a sense of patriotism were found to improve their coping with PTSD symptoms (Nir et al., 2013).

Islamic beliefs also provide patients with a framework for making sense of their experiences and coping with mental health challenges. For example, seeking solace in prayer, reciting religious texts, or engaging in spiritual practices may be integral to healing and recovery. A comprehensive review by Koenig and Al-Shohaib (2019) found that practicing Islam through activities such as reading and reciting the Qur’an, regular prayer, devout belief, strict adherence to Qur’anic teachings, and a supportive community can alleviate stress and promote mental health, overall well-being, and happiness. Harandy et al. (2009) studied spirituality among Muslim breast cancer survivors in Iran. The majority attributed their cancer to the will of God and actively engaged in treatment. The authors argued that spirituality provided cancer patients with psychological support that motivated them to commit to treatment and that this contrasts with Western culture, where a belief in an external health locus of control results in a passive attitude toward screening and treatment (Daaleman et al., 2008). Among Jordanian Muslim men with coronary artery disease, faith enhanced their inner strength, acceptance of their illness, hope, and finding meaning in life (Nabolsi and Carson, 2011).

The biopsychosocial-spiritual model can guide this aspect of religion and include faith-informed interventions that work in conjunction to appropriately address cultural concerns while reforming people’s misconceptions of the relation between faith and the elements of illness, struggle, autonomy, and fate. In encouraging a return to God, forgiveness can help establish empowerment. A coping mechanism based on forgiveness can yield psychological resilience and regulation (Worthington and Scherer, 2004). Positive religious coping beliefs, such as forgiveness, appear to result in a more positive outcome for patients than negative beliefs, such as punishment from God (Lucchetti et al., 2021). The importance of focusing on forgiveness in the experience of psychiatric illnesses can be used to build community, understanding, and empathy, whereas constructs of punishment facilitate seclusion, stigmatization, and suffering (Worthington and Scherer, 2004).

## Evil eye, possession, and black magic

In the Middle East, religiosity is deeply intertwined with cultural and social norms, shaping individuals’ perceptions of mental health and illness, as well as how individuals interpret their experiences and seek support for mental health concerns (Al-Krenawi and Graham, 2000). Supernatural entities govern one component of the Muslim culture’s conceptualizations of mental illness. An Islamic view exists in which it is believed that mental health problems are a result of interventions from the Jinn (genie), black magic, and the effects of the evil eye (Rassool, 2015, 2018). It postulates that these factors would especially affect those who have lost faith and attach more value to the present world (Haque, 2004). A study by Rassool (2018), for example, found that the majority of Muslim patients perceive their symptoms as manifestations of divine punishment, spiritual distress, or supernatural forces such as the evil eye or black magic. Explicitly, the evil eye is believed to cause harm or misfortune via jealousy or envy. At the same time, black magic is perceived as a malevolent force that can adversely impact one’s well-being (Rassool, 2018). Both Al-Ashqar (2003) and Al-Habeeb (2003) also found that symptoms such as anxiety, hyperactivity, altered consciousness, and psychotic disturbances are regularly attributed to the evil eye, magic, or jinn possession within the Muslim community.

Cultural beliefs in the supernatural (evil eye, possession, and black magic) remain abundant within the Middle Eastern population. Variations in the samples studied for research make comparisons challenging. Some studies within the Middle East focused on specific diagnoses or locations and thus cannot be generalized at a population level. In the UAE, mental illness was attributed to the evil eye in 26%, black magic in 25%, and possession in 10% of the population studied (Adel et al., 2023). Slightly higher figures were found in Qatar, where 38.7% of the studied population attributed mental illness to possession (Ghuloum et al., 2010). Among patients with obsessive-compulsive disorder in Egypt, 78.4% reported possession, 64.9% black magic, and 45.9% evil eye as the cause (Okasha et al., 2021). These interpretations are important to consider within the context of faith, as religiosity often guides patients toward seeking support from religious leaders, faith healers, or traditional healers alongside or in place of seeking professional mental health care (El-Islam, 2009; Amro et al., 2019).

## Faith healers

Given the role religion plays in the Middle Eastern understanding of mental illness, people with lived experience often opt to seek care from faith healers rather than mental health services. The existence of several barriers to accessing mental healthcare, including stigma, available expertise, and accessibility, further promotes traditional healing (Gearing et al., 2013). The two terms are often used interchangeably, though in practice they are not. Faith healers use religious-based healing practices, while traditional healers may include alternative practices that are not always religiously guided.

Faith healers are commonly consulted in the Middle East for spiritual healing of mental health issues (Al Shelali et al., 2024), and patients may further seek traditional Islamic remedies, such as recitation of Quranic verses or spiritual rituals, alongside or in place of conventional treatment (Utz, 2013). Muslims may utilize traditional healing practices and modern medical interventions in health-seeking behaviors. Traditional remedies, such as herbal treatments, cupping therapy (hijama), and spiritual healing (ruqyah), are often sought alongside conventional medical care, reflecting a culturally-driven approach to health and well-being (Mayberry, 2022).

The option of traditional healing is understandable, considering the high belief that mental illness is a punishment caused by an evil eye. Traditional healers may better understand the patient’s symptoms through a shared cultural background and provide patients with a socially more acceptable interpretation and treatment (Khoury et al., 2024). They are, therefore, often the first resort in seeking treatment. The rate of seeking traditional healers varies from 34.1% in Tunisia to 70.5% in Saudi Arabia. In the United Arab Emirates (UAE), 28% visited a traditional healer before consulting mental health providers, the majority for depression, and following advice from a friend or family member. Women and those with a high school education or lower were more likely to seek traditional healing. Other studies from the UAE report a wider range, from 44.8% for all diagnoses to 61.7% for bipolar disorder (Adel et al., 2023). In Qatar, 39.1% of the sample studied reported they would visit a healer if they had mental health concerns, and the majority were the younger generation aged below 30 (Ghuloum et al., 2010; Amro et al., 2022). In some studies, e.g., from Egypt, the rate of seeking traditional healers, although it remained high, varied according to diagnosis (Okasha et al., 2021). It is known that cultural and religious backgrounds influence the nature of symptoms presenting in mental illness and the content of psychotic experiences. Thus, some authors found that psychopathology rather than diagnosis is more relevant to the choice of traditional healing over conventional medicine (Adel et al., 2023).

Patients’ religiosity can influence their treatment preferences and decision-making regarding mental health care. Some patients may prioritize religious or spiritual interventions, such as counseling with a religious leader, participation in religious rituals, or integrating faith-based practices into therapy. Mental health care providers must thus acknowledge the role of faith healers and traditional remedies in patients’ help-seeking behaviors and work conjointly with them within ethical boundaries to ensure holistic care. Further, mental health care providers must respect patients’ preferences and collaborate to develop treatment plans that align with their religious beliefs and values, fostering a therapeutic alliance built on trust, respect, and cultural sensitivity (Nyashanu et al., 2022). Religiosity can impact patients’ adherence to treatment interventions prescribed by mental health care providers. Patients may prioritize religious practices and rituals over medication or therapy, leading to non-adherence to treatment plans. Conversely, integrating religious and spiritual beliefs into treatment interventions may enhance patients’ adherence and engagement with therapy. Mental health care providers who acknowledge and respect patients’ religious beliefs can build trust and rapport, fostering collaboration in the treatment process (Smolak et al., 2013).

## Suicide

In Islamic teachings, life is characterized as a gift. Suicide is condemned and seen as a sin; this characterization influences attitudes toward suicide among Muslims (Stack and Kposowa, 2011). However, there is an agreement among religious and Muslim scholars that people with severe mental illness are not responsible for such acts and should be treated in a supportive and human approach (Shoib et al., 2022). The rates of suicide in the Middle East and North Africa region vary, and many believe it is underreported because of the stigma, as suicide is culturally perceived as a taboo (Arafat, 2022; Mahesar et al., 2023).

Literature showing an inverse relationship between spirituality/religiosity and suicidal behavior is well established: the higher the religious affiliation of an individual, the lower their suicide risk. Religion is protective against suicide attempts and completed suicide, but not suicidal ideation (Wu et al., 2015; Lawrence et al., 2016). Community and congregation are integral to the Muslim community, observed through Friday prayer. This aspect of religious involvement and support is based on highlighting Islamic virtues and teachings, which promote hope and purpose (Koenig, 2009; Ghotbi et al., 2019). Viewing suicide as a taboo in the Muslim community is likely to influence the clinician’s comfort in enquiring about suicidal and self-harming ideations in patients. In a study exploring clinical research coordinators’ (CRC) experience with screening for suicidality in patients with schizophrenia, the CRCs reported patients often reacted defensively and used religious phrases reflecting the forbidden status of suicide in Islam. Patients were frequently not comfortable talking about the subject and denied suicidal thoughts even when present. Both CRCs and patients reported that religion may contribute to minimizing the disclosure of suicidal ideation. Patients described how suicide can have implications for the entire family or tribe (Amro et al., 2022). On the other hand, research from the region also suggests that improving the knowledge about suicide and the relevance of religion can improve the reporting and decrease the stigma associated with suicide in Arab countries (Fekih-Romdhane et al., 2023b).

## Conclusion

Psychiatrists and other mental health care providers need to have a better understanding of the patients’ religiosity and how it affects their understanding of their condition, help-seeking behavior, and adherence to treatment interventions. In a region where religion is a fundamental aspect of daily life, working more collaboratively with religious scholars and faith healers is crucial. While accepting religion and spirituality may not differ much in an Islamic context, there is a need to develop models of spiritual care that guide individualized, patient-centered mental health service delivery. Understanding the religiosity of patients is vital for psychiatrists and mental health care providers to deliver holistic, patient-centered care that acknowledges and respects the diverse religious and spiritual dimensions of individuals’ lives. By fostering open dialogue, cultural humility, and collaboration, providers can create therapeutic environments that honor patients’ religiosity while addressing their mental health needs with compassion and expertise. Psychiatrists must respect their patients’ religious or spiritual beliefs, which often play a significant role in their overall well-being. While these beliefs may seem unusual or unhealthy, psychiatrists and mental health practitioners must approach them with sensitivity and an open mind. According to Koenig (2009), in contexts where patients’ beliefs appear to be helping them cope, it may be appropriate for the psychiatrist to offer support. However, it is essential to proceed with caution and avoid taking sides until the psychiatrist has a full understanding of the patient’s psychological condition and personality. Scholars such as Koenig (2009) widely advocate a neutral and respectful approach in the literature.

## Data availability statement

The original contributions presented in the study are included in the article/supplementary material; further inquiries can be directed to the corresponding author.

## Author contributions

SG: Conceptualization, Data curation, Methodology, Project administration, Supervision, Writing – original draft, Writing – review & editing. HA-T: Data curation, Writing – original draft. HA-A: Funding acquisition, Supervision, Writing – review & editing.
